# Activated hepatic stellate cells play pivotal roles in hepatocellular carcinoma cell chemoresistance and migration in multicellular tumor spheroids

**DOI:** 10.1038/srep36750

**Published:** 2016-11-17

**Authors:** Yeonhwa Song, Se-hyuk Kim, Kang Mo Kim, Eun Kyung Choi, Joon Kim, Haeng Ran Seo

**Affiliations:** 1 Cancer Biology Research Laboratory, Institut Pasteur Korea, 16, Daewangpangyo-ro 712 beon-gil, Bundang-gu, Seongnam-si, Gyeonggi-do, 13488, Korea; 2 Laboratory of Biochemistry, Division of Life Sciences, Korea University, 145, Anam-ro, Seongbuk-gu, Seoul, 02841, Korea; 3 Division of Gastroenterology and Hepatology, ASAN Medical center, Olympic-ro 43-gil, Songpa-gu, Seoul, 05505, Korea; 4 Division of Radiation Oncology, ASAN Medical center, Olympic-ro 43-gil, Songpa-gu, Seoul, 05505, Korea

## Abstract

Most Hepatocellular carcinoma (HCC) are resistant to conventional chemotherapeutic agents and remain an unmet medical need. Recently, multiple studies on the crosstalk between HCC and their tumor microenvironment have been conducted to overcome chemoresistance in HCC. In this study, we formed multicellular tumor spheroids (MCTS) to elucidate the mechanisms of environment-mediated chemoresistance in HCC. We observed that hepatic stellate cells (HSCs) in MCTS significantly increased the compactness of spheroids and exhibited strong resistance to sorafenib and cisplatin relative to other types of stromal cells. Increased collagen 1A1 (COL1A1) expression was apparent in activated HSCs but not in fibroblasts or vascular endothelial cells in MCTS. Additionally, COL1A1 deficiency, which was increased by co-culture with HSCs, decreased the cell-cell interactions and thereby increased the therapeutic efficacy of anticancer therapies in MCTS. Furthermore, losartan, which can inhibit collagen I synthesis, attenuated the compactness of spheroids and increased the therapeutic efficacy of anticancer therapies in MCTS. Meanwhile, activated HSCs facilitated HCC migration by upregulating matrix metallopeptidase 9 (MMP9) in MCTS. Collectively, crosstalk between HCC cells and HSCs promoted HCC chemoresistance and migration by increasing the expression of COL1A1 and MMP9 in MCTS. Hence, targeting HSCs might represent a promising therapeutic strategy for liver cancer therapy.

Worldwide, hepatocellular carcinoma (HCC) is one of the human cancers with a high mortality rate despite its early diagnosis in patients and improvements in therapeutic technology. HCC accounts for up to 90% of all primary liver cancers and represents a major health problem^1^,^2^. Chronic infection by hepatitis B and C and chronic alcohol consumption are major causes, as well as metastasis from tumors elsewhere in the body. Because only 10–20% of liver cancers can be surgically removed, the prognosis for the disease is very poor^3^. The cumulative 3-year recurrence rate remains high, approximately 80% after resection with a curative aim, and usually results in a high rate of mortality^4^. Moreover, most HCC exhibit resistance to conventional chemotherapeutic agents. Therefore, the development of an effective HCC treatment strategy remains an unmet medical need^5^.

Accordingly, researchers have aimed to derive target genes and drug candidates for HCC; however, the development of targeted drugs has not yet significantly improved outcomes^5^,^6^. Lately, the paradigm in cancer biology has shifted from the study of the genetics of tumor cells alone to the complicated crosstalk between cancer and the tumor microenvironment (TME)^7^,^8^,^9^. The TME is the cellular environment in which the tumor exists, including the surrounding blood vessels, immune cells, fibroblasts, other cells, signaling molecules, and the extracellular matrix (ECM). Recent studies have shown that the stromal cells in HCC have a dynamic and flexible function in tumor proliferation, invasion, and metastasis, and that the cells of the TME can regulate the response of cancer cells to chemotherapy^10^,^11^,^12^.

Hepatic stellate cells (HSCs) play critical roles in diverse aspects of liver physiology, including liver organogenesis, regeneration, and HCC. HSCs are found in the space of Disse between the sinusoidal endothelial cells and hepatic epithelial cells^13^. HSCs are quiescent and accumulate numerous vitamin A lipid droplets in a healthy liver^14^,^15^. When the liver is wounded by viral infection or hepatic toxins, HSCs undergo a phenotypic transformation from quiescent cells to activated myofibroblast-like cells, and secret diverse cytokines, growth factors, and EMC proteins to protect the liver. Hallmarks of HSC activation are reduced levels of intracellular lipid droplets, increased expression of α-smooth muscle actin (α-SMA) and ECM production, as well as morphological changes^16^,^17^,^18^. Additionally, the interaction between HCC and activated HSCs forms a pro-angiogenic microenvironment by the overexpression of VEGF-α and matrix metallopeptidase 9 (MMP9)^17^,^19^,^20^.

ECM-related proteins in the TME play important roles in liver function in health and disease. Abnormal ECM composition and structure in solid tumors are the major obstacles for the penetration of anticancer drugs. Among ECM proteins, collagens are the most abundant structural protein in the liver. A disproportionate concentration of collagens results in altered cell phenotypes and architectural distortion with abnormal blood flow in the liver. Moreover, a high collagen content is a key barrier for interstitial drug penetration among ECM-related proteins^21^,^22^,^23^ and thereby reduces the efficacy of chemotherapeutics. Because HCC is developed from chronically damaged tissue that contains a large amount of inflammation and fibrosis, further knowledge of the crosstalk between HCC and their TME is essential for achieving a better understanding of tumor development, progression, and chemoresistance in HCC.

In order to recapitulate the interplay between HCC and its microenvironment, the multicellular tumor spheroid model (MCTS) has emerged as a powerful method for mirroring tumor complexity and heterogeneity enhancement in anticancer research^24^. *In vitro*, MCTS has been used in cancer research as an intermediate model between an *in vitro* cancer cell line culture system and an *in vivo* tumor, because MCTS can closely mirror the three-dimensional (3D) cellular context and therapeutically relevant pathophysiological gradients of *in vivo* tumors, such as pH and oxygen gradients, penetration rate of growth factors, and the distribution of proliferating/necrotic cells^25^,^26^,^27^. In particular, liver cells performed a greater number of liver cell functions, including albumin and urea synthesis, bile secretion, and cell polarization in a 3D versus 2D culture system^28^,^29^,^30^.

In this study, we examined the effect of crosstalk between tumors and their microenvironment on chemoresistance and migration using HCC-MCTS models and found that the interaction between HCC cells and HSCs could facilitate the compactness of HCC spheroids via the accumulation of collagen 1A1 (COL1A1), thereby leading to chemoresistance in HCC.

## Results

### The interaction between stromal cells and HCC cells facilitates the compactness of tumor spheroids

HCCs strongly enhance resistance to chemotherapy together with their TME, because the stromal cells in solid tumors contribute to many aspects of carcinogenesis, cancer progression, and chemoresistance^12^.

To model tumor complexity and heterogeneity *in vitro,* we formed MCTS with HCC cell lines and stromal cells representing LX2 (human hepatic stellate cells, HSCs), WI38 (human fibroblasts), and HUVECs.

**Figure 1:**
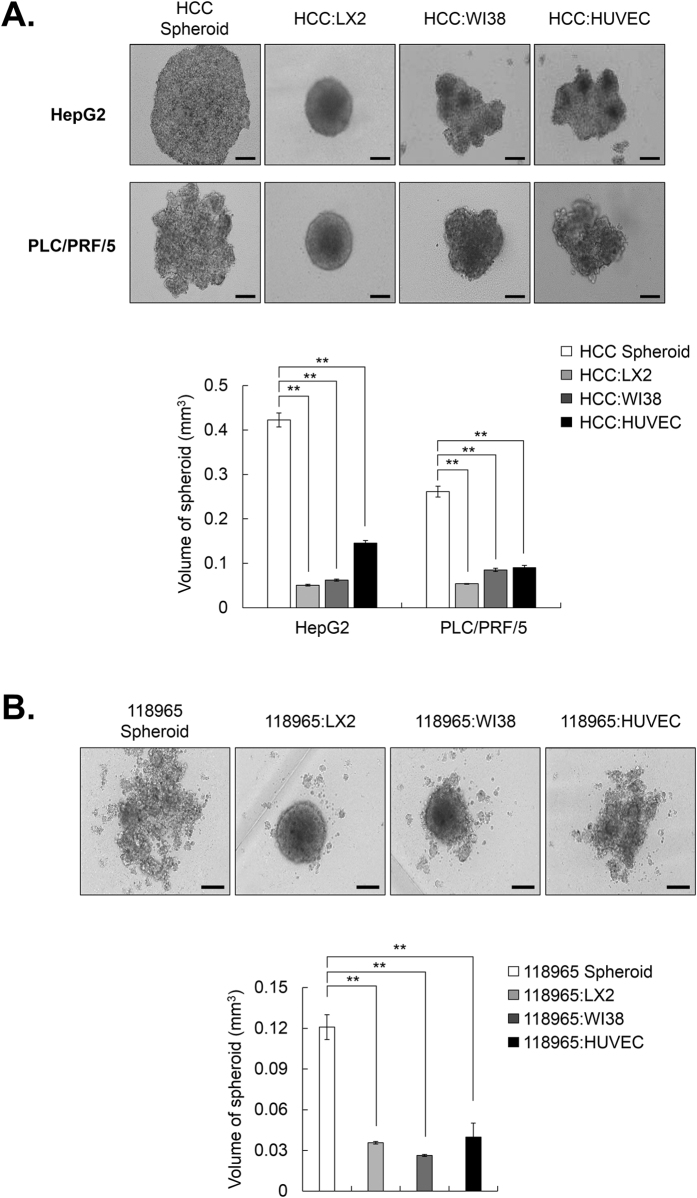
Human hepatic stellate cells (HSCs; LX2) enhance the compactness of loosely aggregated spheroids from HCC cell lines and primary HCC cells. (**A**) HepG2 and PLC/PRF/5 cells, which formed loosely aggregated spheroids alone, were co-cultured with stromal cells (LX2, WI38, and HUVECs) at a 1:1 ratio for 3 days (upper panel). To calculate the volume of spheroids, their long and short diameter were examined (lower panel). (**B**) 118965, which is a primary HCC cell line that formed loosely aggregated spheroids alone, were co-cultured with LX2, WI38, and HUVEC cells at a 1:1 ratio for 3 days (upper panel). Volume of spheroids were analyzed (lower panel). All bright field images of spheroids were obtained using the Operetta^®^ High Content Screening System with a 10× objective. Data are shown as means ± SD from three independent experiments with triplicate. **P < 0.005. Scale bar = 200 μm.

In our previous study, PLC/PRF/5 and HepG2 cells loosely bound to each other and failed to show strong cell cohesion during the progress of spheroid assembly due to the lack of ECM-related components such as collagen I, III, and VI. However, we clearly observed a profound enhancement of spheroid compactness in spheroids with stromal cells and HCC cells relative to HCC-only spheroids [Fig. 1A]. We further ascertained whether 3D co-culture with stromal cells and HCC cells affected the spheroid compactness using primary HCC 118965 cells, which have the capacity to form loosely compacted spheroids. The 3D co-culture with HCC 118965 and stromal cells also exhibited enhanced spheroid compactness [Fig. 1B]. Particularly, co-culture with HCC cells and hepatic stellate cells in MCTS exhibited the most significantly induced enhancement of spheroid compactness compared to HCC-MCTS with WI38 cells or HUVECs.

### Co-culture with HCC cells and HSCs to evaluate chemoresistance in HCC-MCTS

To investigate whether co-culture with HCC cells and stromal cells induces chemoresistance in HCC spheroids, we formed MCTS with HCC cell lines and stromal cells.

**Figure 2:**
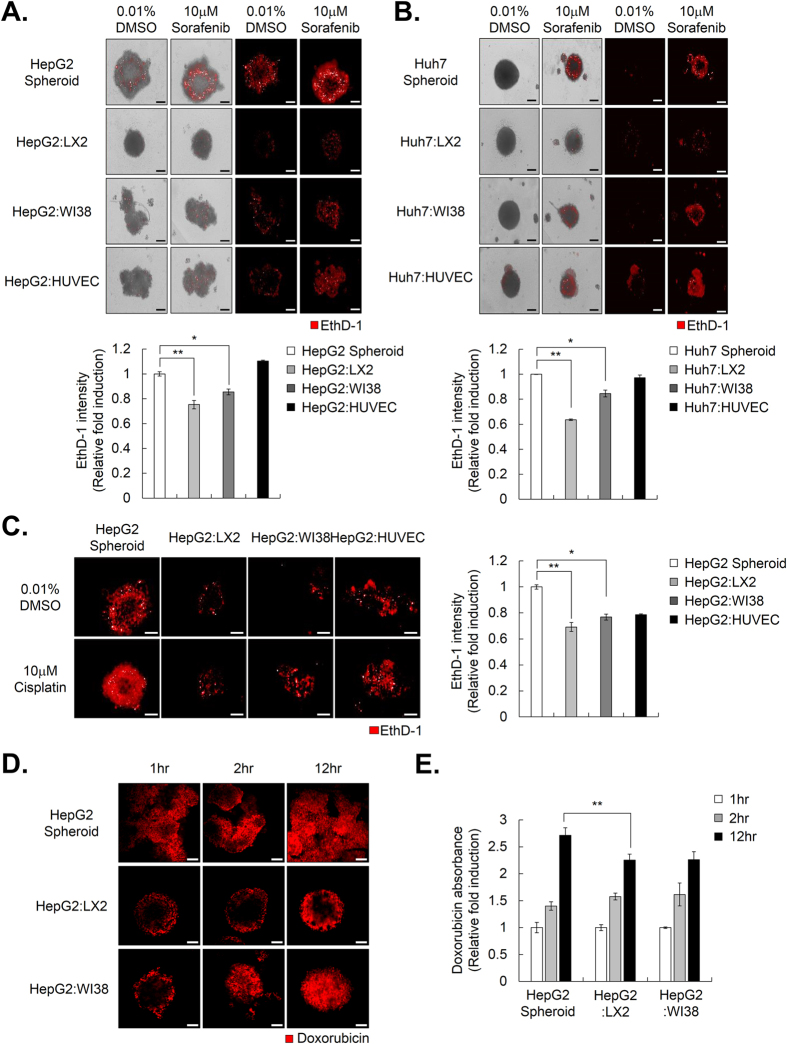
HSCs promote chemoresistance and compactness in multicellular tumor spheroids (MCTS). (**A,B**) Drug sensitivity was measured by EthD-1 (dead cell marker) staining in HepG2 spheroids (**A**) and Huh7 spheroids (**B**), which were co-cultured with or without LX2, WI38, and HUVECs and treated with 10 μM sorafenib for 4 days (upper panel). The intensity of EthD-1 was analyzed and values were normalized to control (0.01% DMSO) (lower panel). Data are shown as means ± SD from three independent experiments with duplicates. (**C**) HepG2 spheroids with or without LX2, WI38, and HUVECs at a 1:1 ratio were treated with 10 μM cisplatin for 4 days (upper panel). Intensity of EthD-1 was examined and values were normalized to control (0.01% DMSO) (lower panel). Data are shown as means ± SD from three independent experiments with duplicates. (**D**) HepG2 spheroids alone or with LX2 or WI38 were treated with 10 μM doxorubicin for the indicated number of hr. The penetration of doxorubicin into the spheroids was measured with LSM with various confocal stacks. (**E**) 10 μM of doxorubicin was treated in HepG2 spheroids alone or with LX2 or WI38 for indicated time. Absorbance of doxorubicin, which was penetrated in each spheroid, was examined. Values were normalized to 1 hr. Data are shown as means ± SD from two independent experiment with duplicate. *P < 0.05, **P < 0.005. Scale bar = 200 μm.

HepG2 tumor spheroids, grown together with LX2, WI38, and HUVEC stromal cells, were incubated with 10 μM sorafenib for 72 hr. Cell death in HCC spheroids, which consisted only of HepG2 cells, and in HepG2-MCTS grown together with HepG2 and HUVECs or WI38 cells, was sufficiently increased by treatment with 10 μM sorafenib, whereas 10 μM sorafenib had less of an effect on cell death in HepG2-MCTS grown together with HepG2 and LX2 cells [Fig. 2A]. When Huh7 cells were used instead of HepG2 cells, sorafenib also significantly induced increases in cell death in Huh7 spheroids and in Huh7-MCTS grown together with HUVECs or WI38 cells compared to Huh7-MCTS grown with LX2 cells [Fig. 2B]. In particular, HCC spheroids grown with LX2 cells exhibited the strongest resistance to sorafenib among the various types of HCC-MCTS. When we examined sensitivity to cisplatin in HepG2-MCTS, HepG2-MCTS grown with LX2 cells also displayed greater resistance to cisplatin compared to HepG2 spheroids and HepG2-MCTS grown with HUVECs or WI38 cells [Fig. 2C].

Next, we compared the efficiency of drug penetration by detecting the distribution of doxorubicin using fluorescence microscopy in HepG2 spheroids and HepG2-MCTS grown with LX2 or WI38 cells. We measured the doxorubicin penetration from the periphery to the central region of the spheroids by multi-layer image acquisition. Interestingly, the differences in doxorubicin penetration were obviously observed between HCC spheroids. Doxorubicin accumulated in the periphery and center of the HepG2 spheroids, whereas doxorubicin only accumulated in the periphery of HepG2-MCTS at 1 hr. Doxorubicin persisted in the periphery of HepG2-MCTS grown with LX2 cells, but not in HepG2-MCTS grown with WI38 cells at 2 h. After incubation with doxorubicin for 12 h, we detected a constant gradient of doxorubicin from the periphery to the central region of the spheroids only in HepG2-MCTS grown with LX2 cells, unlike the other HCC spheroids [Fig. 2D]. To measure the penetrated intracellular doxorubicin, HepG2 spheroids, with or without LX2 cells or WI38 cells, were incubated with doxorubicin for 1, 2, and 12 hr. After collecting the spheroids, they were washed and dissociated to single cells and their absorbance was measured. From this, we confirmed the amount of doxorubicin intercellular in HepG2-MCTS grown with LX2 cells were dramatically lower than HepG2 spheroid, and HepG2-MCTS grown with WI38 cells [Fig. 2E] at 2 and 12 hr. This result prompted us to focus on the crosstalk between HSCs and HCC cells in subsequent experiments because HSCs could increase spheroid compactness, which could be a considerable barrier to drug treatment.

### HSCs in conditioned media (CM) from 3D cultured HCC cells exhibit a myofibroblast-like phenotype

HSCs in the tumor microenvironment undergo a phenotypic transformation from quiescent cells to activated myofibroblast-like cells and could be essential for cancer progression by secreting diverse cytokines, growth factors, and EMC proteins. Because the 3D co-culture of HCC cells and HSCs resulted in strong chemoresistance and increased compactness in HCC-MCTS, we tried to investigate the degree of HSC activation in HCC-MCTS.

**Figure 3:**
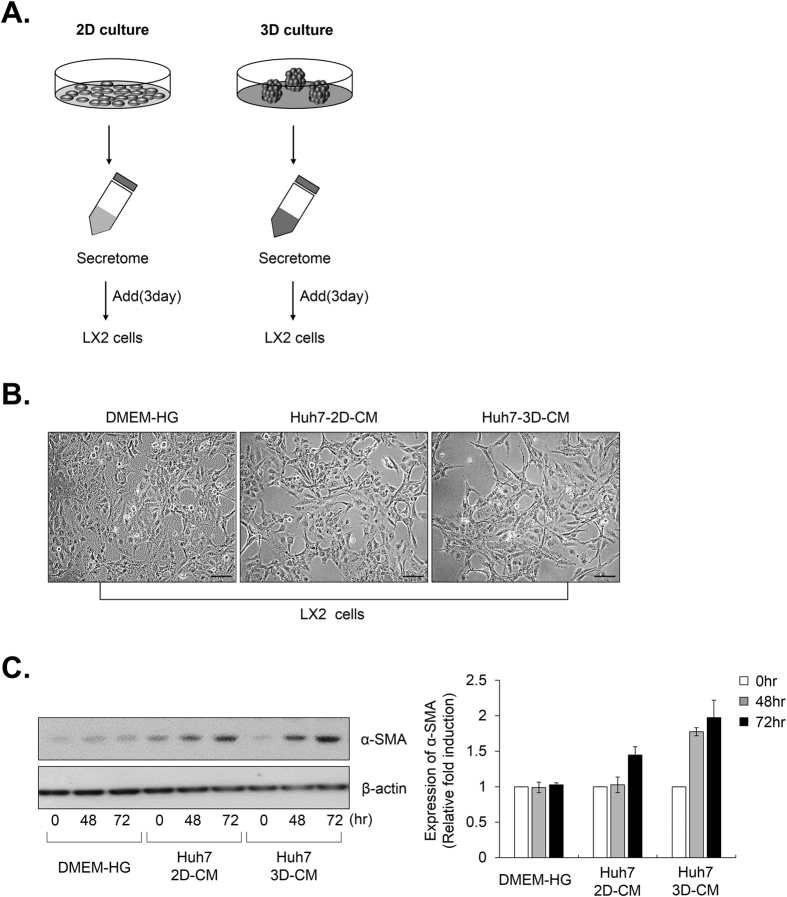
HSCs, which are cultured with conditioned media (CM) from 3D HCC cells, exhibit a myofibroblast-like phenotype. (**A**) Schematic of the CM experiment in (**B,C**). Huh7 cells were cultured under 2D and 3D condition using the same number of cells with the same amount of media. After 3 days, the CM was collected and used to treat LX2 cells for 48 and 72 hr. (**B**) LX2 cells were treated with complete media and CM from 2D and 3D culture for 48 hr. The phenotype of the cells was examined using bright field images. (**C**) LX2 cells that were treated with complete media and CM from the 2D and 3D HCC cells were collected at the indicated times (0, 48 and 72 hr). LX2 lysates were analyzed by western blotting using anti-α-SMA and anti-β-actin (control) antibodies. Values were normalized to β-actin. Data are shown as means ± SD from three independent experiments. Scale bar = 500 μm.

To confirm the activation of HSCs in MCTS, we collected CM from 2D and 3D cultured HCC cells and added both CMs to the LX2 cells [Fig. 3A]. The transformation of LX2 cells to a fibroblast-like morphology was more significant in the CM from the 3D cultured HCC cells than in the CM from the 2D cultured HCC cells [Fig. 3B]. Moreover, we confirmed that the CM from the 3D culture conditions significantly increased the expression of the fibroblast marker α-SMA in a time-dependent manner, compared to CM from the 2D culture conditions [Fig. 3C]. These results indicated that the CM from the 3D culture sufficiently induced LX2 cells to undergo phenotypic conversion from quiescent cells to form myofibroblast-like cells.

### Increased collagen expression by the interaction between HSCs and HCC cells in MCTS

**Figure 4:**
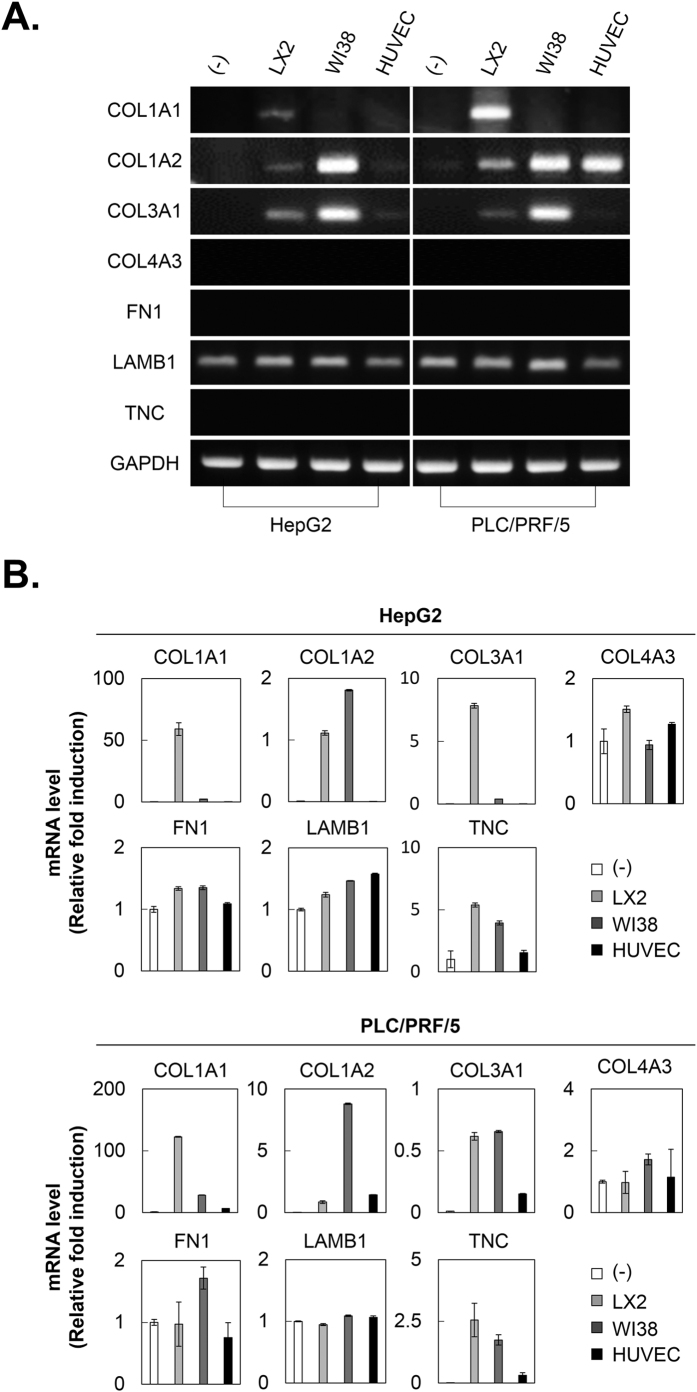
Collagen 1A1 expression is increased by the interaction between HSCs and HCC cells in MCTS. HepG2 and PLC/PRF/5 spheroids were cultured alone or with LX2, WI38, and HUVEC stromal cells at 1:1 ratio for 3 days. After 3 days, mRNA expression in each spheroid was evaluated by (**A**) RT-PCR and (**B**) real-time PCR using collagen and non-collagen-related ECM primers. Values were normalized to GAPDH. Data are shown as means ± SD from two independent experiments with duplicates.

Because activated HSCs in tumor microenvironments can accumulate ECM proteins, we investigated the synthesis of ECM proteins in MCTS. PLC/PRF/5 or HepG2 cell tumor spheroids exhibited a lack of ECM-related components, including collagens (collagen I, III, and IV) and non-collagenous ECM proteins (fibronectin and fibrillin), except laminin. We ascertained whether co-culturing HCC cells and stromal cells could increase the expression of ECM proteins in MCTS. HCC-MCTS grown with WI38 cells displayed increased expression of COL1A2 and COL4A3, and HCC-MCTS grown with HUVECs only induced increases in COL1A2. Particularly, HCC-MCTS with LX2 cells exhibited significantly increased levels of COL1A1, as well as COL1A2 and COL4A3, unlike the other spheroids [Fig. 4]. The expression of non-collagenous proteins was not altered by the co-culturing of HCC cells and stromal cells in MCTS.

### Increased collagen I expression by activated HSCs plays pivotal roles in chemoresistance via the modification of spheroid compactness in HCC-MCTS

**Figure 5:**
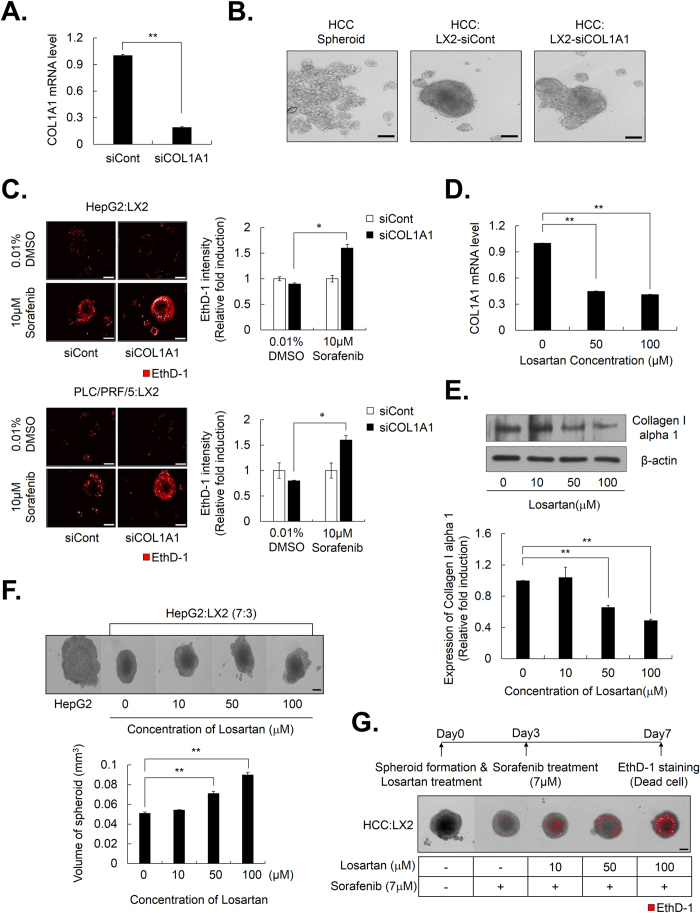
Activated HSCs enhance chemoresistance by increasing the expression of collagen I in MCTS. (**A**) LX2 cells were transfected with siCont and siCOL1A1, and their efficiency was examined by real-time PCR and (**B**) HepG2 cells were cultured with LX2-siCont and COL1A1-depleted LX2 cells (LX2-siCOL1A1) at a 1:1 ratio. The morphology of the spheroids was examined after 3 days. (**C**) Drug sensitivity was investigated in HepG2 and PLC/PRF/5 with LX2-siCont and LX2-siCOL1A1 by EthD-1 staining to detect cell death after 4 days from treating with 10 μM sorafenib. Images were obtained (left) and EthD-1 intensity was analyzed (right). Data represent the mean values ± SD from three independent experiments relative to the value for the control. (**D,E**) Huh7 was treated with losartan with indicated concentration for 3 days for evaluating the COL1A1 mRNA and protein levels through (**D**) real-time PCR and (**E**) western blot. Values were normalized to GAPDH. (**F**) HepG2:LX2 spheroids, which were co-culture at a 7:3 ratio, were treated with the indicated concentration of losartan for 3 days (upper). The volume of spheroids was analyzed (lower). Data represent the mean values ± SD from five independent experiments relative to the value for the control. (**G**) HepG2:LX2 spheroids, which were mixed at a 7:3 ratio, were treated with indicated concentration of losartan for 3 days and then treated with 7 μM sorafenib for an additional 4 days. Seven days after spheroid formation, the spheroids were stained with EthD-1 to detect cell death. *P < 0.05, **P < 0.005. Scale bar = 200 μm.

Because the expression of COL1A1 was actually elevated by the interaction between HCC cells and HSCs in HCC-MCTS, we examined whether there was a change in the compactness of HepG2-MCTS composed of HepG2 and LX2 cells, by depletion of COL1A1 expression. We confirmed that siRNA of COL1A1 (siCOL1A1) efficiently depleted COL1A1 mRNA levels, whereas control siRNA (siCont) treatment did not [Fig. 5A]. To this end, we observed the shape of MCTS with HepG2 and COL1A1-deficient LX2 cells via the transfection of siCOL1A1 into LX2 cells to investigate the effect of COL1A1 inhibition on MCTS compactness. Although MCTS with HepG2 cells and normal LX2 cells exhibited enhanced spheroid compactness, MCTS with HepG2 and COL1A1 deficient LX2 cells did not display tight compactness [Fig. 5B]. As well, morphological changes in the MCTS by depletion of COL1A1 in LX2 cells resulted in greater therapeutic efficacy of sorafenib than cells transfected with control siRNA [Fig. 5C].

Next, we investigated effect of losartan, which can inhibit collagen I synthesis in tumors, on the compactness of HepG2-MCTS composed of HepG2 and LX2 cells. Losartan efficiently decreased the level of COL1A1 mRNA [Fig. 5D] and protein [Fig. 5E] in HCC cells. Interestingly, losartan effectively induced a structural change in the spheroids by reducing the compactness of MCTS with HepG2 and LX2 cells in a dose-dependent manner [Fig. 5F].

We also investigated whether modification of spheroid compactness, which was induced by the inhibition of collagen I synthesis by pretreatment with losartan, could overcome resistance to anticancer therapy in MCTS with HepG2 and LX2 cells. The analysis of cell death revealed that losartan enhanced cell death induced by 7 μM sorafenib in a dose-dependent manner in MCTS [Fig. 5G].

Collectively, these results suggested that increased expression of collagen I by crosstalk between HCC cells and HSCs in MCTS played pivotal roles in chemoresistance in HCC via modification of spheroid compactness.

### Activated HSCs promote HCC migration through the upregulation of MMP9 in MCTS

Because crosstalk between HCC cells and their tumor microenvironment is regarded as critical for the promotion of cell migration, which is the major cancerous process, HepG2-MCTS grown with stromal cells were subjected to a tumor spheroid-based migration assay.

**Figure 6:**
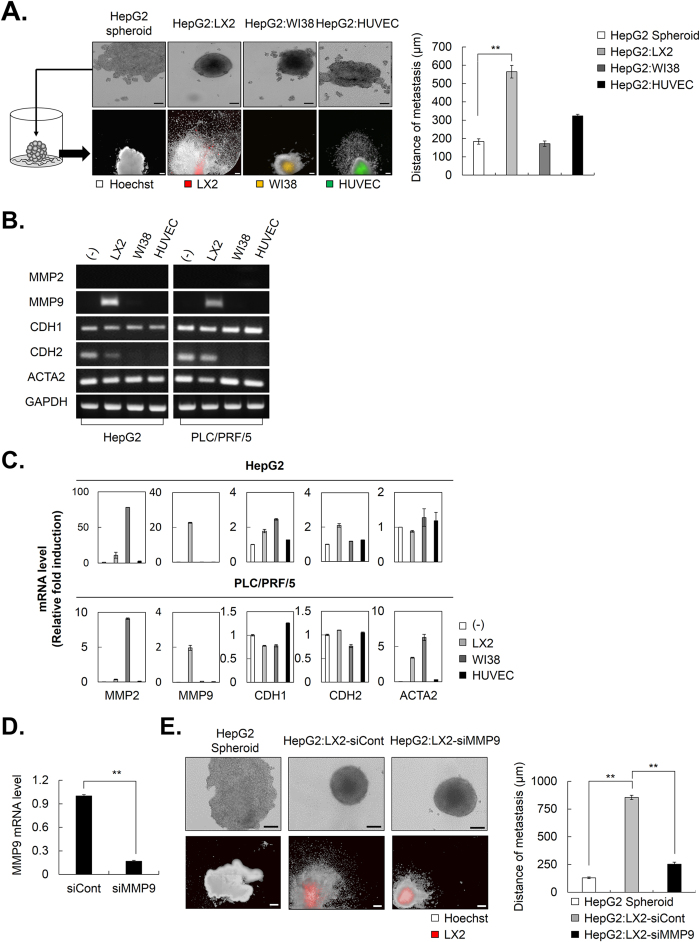
Activated HSCs possess a migration capacity as a result of increased MMP9 in HCC-MCTS. (**A**) HepG2 alone and co-culture spheroids with LX2 cells, which is stained by DiO, WI38 cells stained by DiI, and HUVECs stained by DiD were cultured for 3 days. After spheroid formation, each spheroid was transferred to a collagen I coated plate and cultivated for 4 days. The cells were stained with Hoechst33342 after fixation and the images were obtained (left). The distance from surface of spheroid to boundary of stretched cells was measured (right). (**B,C**) HepG2 and PLC/PRF/5 spheroids were cultured alone or with LX2, WI38, and HUVEC stromal cells at a 1:1 ratio for 3 days. After 3 days, mRNA expression in each spheroid was evaluated by (**B**) RT-PCR and (**C**) real-time PCR using MMP and cadherin primers. Values were normalized to GAPDH. (**D**) LX2 cells were transfected with siCont and siMMP9 and its efficacy was examined by real-time PCR. (**E**) HepG2 alone and co-culture spheroids with LX2-siCont or LX2-siMMP9, which were stained by DiO, were cultured for 3 days. After 3 days, each spheroid was transferred to a collagen I coated plate and cultivated for 4 days. The cells were fixed and stained with Hoechst33342. The images were obtained using the same method (left). The distance from surface of spheroid to boundary of stretched cells was measured (right). Data represent the mean values ± SD from three independent experiments. **P < 0.005. Scale bar = 200 μm.

HepG2-MCTS grown with or without stromal cells were transferred to a collagen I-coated plate and maintained for 4 days. Next, we estimated the distance from the surface of spheroids to boundary of stretched migrating HepG2 cells. The tumor spheroid-based migration assay revealed that HepG2-MCTS grown with LX2 cells significantly facilitated the ability to migrate compared to other MCTS [Fig. 6A].

Non-proteolytic activities of MMPs and cadherins play important roles in cell migration. To assess if migration-related signaling was regulated by co-culturing HSCs in MCTS, we performed RT-PCR and real-time PCR to measure the expression of MMPs and cadherins. The results indicated that activated HSCs specifically increased MMP9, but not MMP2 in MCTS [Fig. 6B,C]. E-cadherin controls cell migration via the generation of strong cell cohesion and is a known mediator of spheroid formation in several cancer types. However, changes in the expression of E- and N-cadherins did not appear to be a predictor of HCC migration in MCTS with HCC cells and LX2 cells. To further evaluate the effect of MMP9 on HCC migration in MCTS, we investigated HCC migration in MCTS with HepG2 and MMP9-deficient LX2 cells via the transfection of siRNA of MMP9 (siMMP9) into LX2 cells. siMMP9 sufficiently depleted expression of MMP9 mRNA [Fig. 6D]. As expected, the MCTS with HepG2 and normal LX2 cells exhibited strong migratory capacity, whereas MCTS with HepG2 and MMP9-deficient LX2 cells did not display [Fig. 6F].

Thus, these results suggested that the interplay between HCC cells and HSCs significantly fostered an environment to facilitate HCC migration in MCTS through increased MMP9 expression.

## Discussion

Heterologous cell types within tumors can actively influence therapeutic responses and shape resistance. Based on these premises, we tried to determine the contribution of distinct components in the TME to tumor progression and chemoresistance by challenge for modeling of tumor complexity and heterogeneity.

In order to establish tumor complexity *in vitro*, we created MCTS with HCC cells and stromal cells, which are HSCs, fibroblasts, and endothelial cells [Fig. 1] and identified that the ruggedness of MCTS was variable depending on the type of stromal cells they contained. Here, we were particularly excited by the realistic reciprocal crosstalk between HCC cells and HSCs in HCC-MCTS, because co-culture with HCC cells and HSCs revealed the enhancement of both the resistance to anticancer drugs, such as sorafenib, cisplatin, and doxorubicin, and the compactness of spheroids in HCC-MCTS [Figs 1 and 2]. HSCs can infiltrate tumor stroma and secret ECM-related proteins, cytokines, and growth factors in tumor environments. Activated HSCs can regulate the ability of HCC cells to migrate and proliferate via the modulation of TGF-β and ECM-related proteins^31^. HSCs also promote chemoresistance in HCC by secreting hepatocyte growth factor (HGF)^32^. Because active HSCs were involved in tumor onset, progression, and chemoresistance, targeting the HSCs might represent a promising therapeutic strategy.

Previous reports have demonstrated that HCC stimulates the activation of HSCs^33^,^34^. HSCs in CM from monolayer cultured Huh7 or HepG2 cells exhibited activation phenotypes and strong migration activity^35^. Because 3D cell culture could better mimic the growth characteristics and microenvironment of solid tumors *in vivo* than a monolayer culture, we conducted comparative studies of the effects of the secretome on the activation of HSCs in HCC-2D and HCC-3D culture conditions. Here, we observed more significant activation of HSCs detected in 3D culture conditions than in the monolayer HCC culture conditions [Fig. 3]. These results indicated that increasing the growth factors in HCC-3D culture conditions appeared to lead to the activation of the HSCs and that activated HSCs led to chemoresistance in the MCTS models. We also ascertained that the paracrine effects of HCC cells on the activation of stromal cells should be investigated in 3D culture conditions.

Activated HSCs are a major ECM-producing HCC cell type. ECM plays an important role in regulating cell morphology, development, migration, proliferation, and cell function. The ECM occupies a small percentage of the volume of the normal liver, whereas many types of tumor tissues are characterized by excessive ECM production or limited ECM turnover. We confirmed that co-culturing HCC cells with HSCs could modulate the expression of collagens in HCC-MCTS. The interaction between HSCs and HCC cells effectively facilitated spheroid compactness, as well as the accumulation of COL1A1, in HCC-MCTS [Fig. 4]. HSCs are the major liver cells capable of upregulating COL1A1 mRNA following a fibrogenic stimulus^36^. Increased COL1A1 mRNA was observed in activated HSCs compared to quiescent HSCs *in vivo*^37^,^38^. Moreover, the half-life of the COL1A1 mRNA was increased about 16-fold in activated HSCs compared to quiescent HSCs^39^.

Studies have shown that high collagen content is a key barrier for drug penetration among ECM-related proteins and thereby reduces the efficacy of chemotherapeutics. When the production of COL1A1 by HSCs was blocked in MCTS, the compactness of MCTS was reduced and the therapeutic efficacy of anticancer drugs was greatly improved [Fig. 5A,B]. Losartan, which is used widely to treat hypertension as an angiotensin II type 1 receptor (AT1) antagonist with noted antifibrotic activity, can decrease collagen I in tumors^40^,^41^,^42^. Here, we also found that the administration of losartan before chemotherapy markedly improved the efficacy of chemotherapeutic drugs by impairing the compactness of MCTSs by disrupting cell-cell contacts [Fig. 5C,D]. Herein, our results revealed that the inhibition of COL1A1 was a good strategy for overcoming cell adhesion-mediated drug resistance in HCC.

Bidirectional interactions between HCC cells and HSCs might function as an “amplification loop” to further enhance metastatic growth in the liver^43^. Under 3D-culture conditions, activated HSCs significantly promoted the metastatic growth of HCC cells compared to fibroblasts and endothelial cells [Fig. 6A]. HSCs express virtually all of the key components required for pathologic matrix degradation and therefore play a key role not only in matrix production but also in matrix degradation. In this study, MMP2 (gelatinase A) and MMP9 (gelatinase B) were the focus of the research, because MMP2 and MMP9 expression is associated with cancer cell invasion and these proteins are elevated in a variety of malignancies^44^,^45^,^46^. The expression of MMP9 is induced in MCTS by co-culture with HSCs; however, the expression of MMP2 was not affected by this culture system [Fig. 6B].

The loss of E-cadherin expression or the gain of N-cadherin expression in carcinomas has long been regarded as a primary reason for the disruption of tight epithelial cell-cell contacts and the release of invasive tumor cells from the primary tumor. However, E- and N-cadherin expression was not changed by the co-culture of HSCs and MCTS. These results showed that activated HSCs facilitated HCC migration by upregulating MMP9 in HCC-MCTS.

Some important questions, including the contribution of distinct components in the TME to tumor progression, the type of signals cancer cells receive from the stromal cells in HCC, and how these signals promote malignant growth, should be solved to enhance our understanding of the realistic communication between HCC and TME. Also, MCTS, which are composed of LX2, WI38, HUVEC and HCC together, to study crosstalk effect between tumor microenvironment and HCC as well as drug screening. In this study, we have challenged the formation of diversiform MCTS to solve these questions. Through this process, we demonstrated that HSCs can promote HCC chemoresistance and migration by increasing COL1A1 and MMP9 expression in MCTS. Hence, targeting the regulation of HSC activation in MCTS could represent a promising therapeutic strategy for overcoming chemoresistance and tumorigenesis in HCC.

## Materials and Methods

### Cell lines and culture conditions

Huh7, PLC/PRF/5, and HepG2 (human HCC cell lines) were obtained from the Korean Cell Line Bank. LX2^47^ (human hepatic stellate cells; HSCs) were provided from Dr. Friedman. WI38 (human fibroblast cells) were purchased from the American Type Culture Collection (AATC; Manassas, VA, USA). Human umbilical vein endothelial cells (HUVEC) were obtained from Lonza (Basel, Switzerland). The cells were maintained at 37 °C in a humidified atmosphere of 5% CO_2_. All HCC cell lines and LX2 were cultured in Dulbecco’s Modified Eagle medium (DMEM; Welgene, Korea) supplemented with 10% fetal bovine serum (FBS; Gibco, Grand Island, NY, USA) and 1× penicillin-streptomycin (P/S; Gibco) (Complete media). WI38 cells were cultivated in Roswell Park Memorial Institute medium (RPMI 1640; Welgene) supplemented with 10% FBS, 1× P/S, and 1× non-essential amino acids (NEAA; Gibco). For HUVECs, endothelial basal medium (EBM) was purchased from Lonza.

### Primary culture of HCC (118965)

Immediately after surgery, a portion of the tumor was immersed in Hanks balanced salt solution (HBSS; Gibco) and transported from the operating room at 0 °C to the laboratory. The specimens were collected under sterile conditions and rinsed 2–3 times with HBSS free of calcium and magnesium to remove blood. After removal of blood, the liver sample was excised, cut into small fragments, gently dispersed and placed in HBSS containing 0.03% pronase, 0.05% type IV collagenase, and 0.01% deoxyribonuclease (DNase, from bovine pancreas) for 20 minutes at 37 °C. The resultant was filtered through a 100 μm-nylon filter (BD Falcon, Franklin Lakes, NJ, USA) and centrifuged at 50 × g for 2 minutes at 4 °C to obtain hepatocytes. The pellet was washed twice in HBSS containing 0.005% DNase. The final cell suspensions were cultured onto collagen-coated T25 flasks (BD Falcon) in F12/DMEM (Gibco), supplemented with 20% FBS, 1% NEAA, 1% glutamine, and 1% P/S at 37 °C in a humidified 5% CO_2_ incubator. The medium was changed 24 hours after seeding to remove dead cells and debris. When confluence reached 70–80%, the cells were re-plated using a 1:1 mixture of DMEM medium and F12/DMEM with supplements. After five passages, the cells were grown DMEM medium supplemented with 10% FBS and 1% P/S. Confluent cells were trypsinized, counted and split 1:3–1:5 at every passage. Once cell lines were maintained over 30 passages, they were collected and stored in liquid nitrogen.

To confirm whether the primary HCC (118965) from hepatocytes, we performed immunostaining of Hep Par-1 (Dako, Denmark A/S, Denmark; M7158, Clone OCH1E5), AFP (Dako; A0008) and albumin (Dako, A0001) which are hepatocytes-specific markers and quantify the expression of markers. In addition, AFP and albumin gene expression levels were analyzed by real-time PCR to assess liver-specific functional competence (Fig. S1).

### Ethics approval and Consent to participate

The study was conducted in accordance with the Declaration of Helsinki principles. It was approved by the Human Research Ethics Committee of ASAN medical center. The institute review board in ASAN medical center complies with the related laws such as ICH, KGCP or bioethics and safety act. Written informed consent for the use of tissues for research was taken from patients at the time of procurement of tumor specimens.

### High-content screening and imaging technology

All bright-field and fluorescence images were obtained using the Operetta^®^ High-Content Screening System (HCS; Perkin Elmer, Waltham, MA, USA) with a 10× objective for spheroids and a 40× objective for monolayers. The fluorescence images were captured according to the optimal excitation and emission wavelengths of each probe.

### Generation of tumor spheroid and drug treatment

To generate spheroids, cells suspended in complete medium were seeded at a density of 6 × 10^3^ cells/well in 96-well round-bottom ultra-low attachment microplates (Corning B.V. Life Sciences, Amsterdam, Netherlands). The plates were incubated for 3 days at 37 °C in a humidified atmosphere of 5% CO_2_. To generate the MCTS with various cell types, HCC cells and stromal cells (LX2, WI38, and HUVECs) were mixed at a 1:1 ratio. For losartan combination study, HCC:LX2 spheroid was mixed at 7:3 ratio. For drug treatment, the cells were seeded with or without losartan (10, 50, or 100 μM; Sigma-Aldrich, St Louis, MO, USA) for 3 days and then with 7 or 10 μM of sorafenib (Santa Cruz Biotechnology, Santa Cruz, CA, USA) and 10 μM of cisplatin (Sigma-Aldrich) for another 4 days. The volume of spheroid was determined using the following formula: (L x I^2^)/2, where L = long diameter and I = short diameter of spheroid^48^.

### Cell death detection in spheroid

Spheroid cell death was detected using the cell-impermeant viability indicator ethidium homodimer-1 (EthD-1; Invitrogen, Eugene, OR, USA). EthD-1 is a high-affinity nucleic acid stain that fluoresces weakly until binding to DNA and emits red fluorescence (excitation/emission maxima ~528/617). Spheroids were incubated in 4 μM EthD-1 in complete medium for 30 min in a 37 °C incubator and images and the intensity of EthD-1 was obtained using the HCS system

### Drug penetration in spheroid

HepG2 spheroids, alone or with LX2 or WI38 cells at a 1:1 ratio were formed for 3 days. After formation, the spheroids were treated with 10 μM doxorubicin (Sigma-Aldrich) for 1, 2, and 12 hr. For the image acquisition, each spheroids were fixed in 4% paraformaldehyde (Biosesang, Korea) for 30 min, and washed with Dulbecco’s phosphate-buffered saline (DPBS, Welgene) twice. The auto fluorescence of doxorubicin was detected using an inverted Zeiss laser scanning microscope (LSM) with a 100 W HBO mercury light source equipped with a 530 to 560 nm excitation and a 573 to 647 nm emission filter set. Spheroid images were captured in a 20 μm stack using 10× objectives. For measuring the absorbance of doxorubicin inside the spheroids, spheroids washed with DPBS twice in 1.5 ml tube after doxorubicin treatment for 1, 2, and 12 hr. After washing, each spheroid was transferred to 96-well and treated with 0.05% trypsin for 30 min at 37 °C incubator. After 30 min, spheroid in each well was pipetted smoothly until being dissociation to single cells. Using the 560 nm filter of spectrophotometer (Enspire, Perkin Elmer), absorbance of doxorubicin was measured.

### Conditioned medium experiment

Huh7 were cultured in 2D or 3D conditioned medium (CM) using the same number of cells and amount of medium for 3 days. CM were collected when cells were reached 70–90% confluence in case of 2D culture condition. The CM were obtained and filtered with a 20 μm pore filter (Millipore, Billerica, MA, USA) to eliminate debris. All CM from 2D and 3D was mixed with fresh media at 8:2 ratio, and LX2 complete medium was used as a control. LX2 cells were seeded at a density of 5 × 10^5^ cells in a 100-mm^2^ dish and treated with 5 ml of 2D- or 3D-CM for 48 and 72 hr without media change. The morphology of cells was examined with a Zeiss microscope and the cell lysates were collected for western blotting.

### Migration of spheroid

Before generating the spheroids, LX2, WI38, and HUVECs were stained with DiD (633 nm), DiI (546 nm), and DiO (488) (Invitrogen) individually at a 1:500 ratio in complete media for 20 min in a 37 °C incubator in suspension. After washing with DPBS, the spheroids were formed for 3 days and each spheroid was relocated to a collagen I-coated 384-well plate (Greiner Bio-one, Monroe, NC, USA) and cultured for 4 days in complete media. Medium was changed once at 2 days after relocating. After 4 days, the cells were fixed in 4% paraformaldehyde (Biosesang) for 30 min at room temperature (R.T.) and washed with DPBS twice. The fluorescence images were captured according to the optimal excitation and emission wavelengths of each probe. To capture the entire images, 25 image fields, starting at the center of the well, were collected from each well using HCS system with a 10× objective. The distance from surface of spheroid to boundary of stretched cells was determined using the HCS software.

### Short interfering RNA (siRNA) transfection

siRNA probes were designed by and purchased from Dharmacon (Lafayette, CO, USA). LX2 cells were seeded and the medium was replaced with Opti-MEM (Gibco) without FBS and antibiotics when the cell density reached 40–50%. The siCOL1A1 sequences were as follows: COL1A1 #1, 5′-GCAAGACAGUGAUUGAAUA-3′; #2, 5′-GGAAUUCGGCUUCGACGUU-3′; #3, 5′-CCAGCUGUCUUAUGGCUAU-3′; #4, 5′-GCUCGAGGAUUGCCCGGAA-3′, MMP9 #1, 5′-GCAUAAGGACGACGUGAAU-3′; #2, 5′-GGACCAAGGAUACAGUUUG-3′; #3, 5′-GCGCUCAUGUACCCUAUGU-3′; #4, 5-GAACCAAUCUCACCGACAG-3′. Cells were co-trnasfected with the four siRNAs targeting COL1A1 (siCOL1A1), MMP9 (siMMP9) and scramble (siCont) for 24 hr using Lipofectamine^®^ 2000 (Invitrogen).

### Statistical analysis

All experiments were performed in duplicate. The results are expressed as the mean ± standard deviation (SD). Statistical analysis was performed using Student’s t-test.

## Additional Information

**How to cite this article**: Song, Y. *et al.* Activated hepatic stellate cells play pivotal roles in hepatocellular carcinoma cell chemoresistance and migration in multicellular tumor spheroids. *Sci. Rep.* **6**, 36750; doi: 10.1038/srep36750 (2016).

**Publisher’s note**: Springer Nature remains neutral with regard to jurisdictional claims in published maps and institutional affiliations.

## Supplementary Material

Supplementary Information
