# Supplementary material for: Activated hepatic stellate cells play pivotal roles in hepatocellular carcinoma cell chemoresistance and migration in multicellular tumor spheroids
Source: Sci Rep. 2016 Nov 17;6:36750. doi: 10.1038/srep36750 (PMC5113076; doi:10.1038/srep36750)

Supplementary Information

Activated hepatic stellate cells play pivotal roles in hepatocellular carcinoma cell chemoresistance and migration in multicellular tumor spheroids

Yeonhwa Song[1,4], Se-hyuk Kim[1], Kang Mo Kim[2], Eun Kyung Choi[3], Joon Kim [4*] and Haeng Ran Seo[1*]

Author Affiliations:

[1]Cancer Biology Research Laboratory, Institut Pasteur Korea, 16, Daewangpangyo-ro 712 beon-gil, Bundang-gu, Seongnam-si, Gyeonggi-do, 13488, Korea, [2]Division of Gastroenterology and Hepatology, ASAN Medical center, Olympic-ro 43-gil, Songpa-gu, Seoul, 05505, Korea, [3] Division of Radiation Oncology, ASAN Medical center, Olympic-ro 43-gil, Songpa-gu, Seoul, 05505, Korea, [4] Laboratory of Biochemistry, Division of Life Sciences, Korea University, 145, Anam-ro, Seongbuk-gu, Seoul, 02841, Korea.

*Corresponding author: Haengran Seo, Cancer Biology Research Laboratory, Institut Pasteur Korea, 16, Daewangpangyo-ro 712 beon-gil, Bundang-gu, Seongnam-si, Gyeonggi-do, 13488, Korea ; Tel: +82-31-8018-8300; E-mail: shr1261@ip-korea.org
or Joon Kim, Laboratory of Biochemistry, Division of Life Sciences, Korea University, Seoul 02841, Korea. ; E-mail: joonkim@korea.ac.kr

Supplementary information

Reverse transcription-polymerase chain reaction (RT-PCR) and real-time polymerase chain reaction (qRT-PCR)

Total RNA was isolated from cells using TRIzol® (Invitrogen, Eugene, OR, USA) according to the manufacturer's instructions. The reaction mixtures were comprised of RT buffer (Bio Basic, Amherst, NY, USA), dNTP solution (Bio Basic), RNasin® Inhibitor (Promega, Madison, WI, USA), oligo $(dT)^{15}$ primer (Bioneer, Daejeon, Korea), total RNA, and M-MLV reverse transcriptase (Invitrogen). The reaction mixtures were incubated at 37℃ for 1 h and the transcription reaction was terminated by heating the mixture to 95℃ for 5 min and then rapidly cooling it on ice. The number of PCR cycles used was 30 for all reactions. The PCR products were then separated by 2% agarose gel electrophoresis and visualized with 5× Loading Star (Dynebio, Seoul, Korea). For real-time PCR, the mixture composed of cDNA, SYBR Green master mix (Applied Biosystems, Waltham, MA, USA), primers and DEPC, were performed using a StepOnePlus real-time PCR system (Applied Biosystems). The reactions were incubated in a 96-well optical plate at 95℃ for 10 min, followed by 40 cycles of 95℃ for 15s and 60° for 10 min. The threshold cycle (CT) is defined as the fractional cycle number at which the fluorescence passes the fixed threshold. CT values were normalized to GAPDH, and calculated according to the mathematical model R = 2−ΔΔCT, where Δ CT = CTtarget gene − CT_{GAPDH}, and ΔΔ CT = ΔCTtest − ΔCTcontrol. All real-time PCR was performed in triplicate, and the data are presented as the mean ± SD. All primers (Supplementary Table S1 and S2) were designed and purchased from Bioneer.

Polyacrylamide gel electrophoresis (PAGE) and western blot analysis

Cells were solubilized in lysis buffer (3M, Maplewood, MN, USA), boiled for 5 min, and 10 μg of protein for β-actin, 30 μg of protein for α-SMA and 50 μg of protein for Collagen IA1 were loaded in each wells equally on 10% SDS-PAGE gels. After electrophoresis, the proteins were transferred onto a polyvinylidene difluoride (PVDF) membrane (Millipore, Billerica, MA, USA) and blocked with 5% skim milk for 30 min at R.T. After blocking, the PVDF membranes were incubated with anti-α-SMA (Abcam, Cambridge, USA; ab5694). Collagen IA1 (Novus Biologicals, Littleton, Colorado, USA; NB600-408) and β-actin

(Sigma-Aldrich; A5441; Clone AC-15) for 16 h at 4℃. After washing, the blots were incubated with horseradish peroxidase-conjugated secondary antibody (Cell Signaling Technology, Danvers, MA, USA) at a 1:10000 dilution, and specific bands were visualized by enhanced chemiluminescence (ECL; Thermo Scientific, Waltham, MA, USA) and recorded on X-Omat AR films (Eastman Kodak Co., Rochester, NY, USA).

Supplementary Table S1. Primer sequence for RT-PCR.

Gene	Primer sequence	
	Forward (5'-3')	Reverse (3'-5')
COL1A1	CGCTGGTTTCGACTTCAGCT	ACATTGGCATCATCAGCCCG
COL1A2	CTGGCCCCAATGGATTTGCT	CCGTTTTCACCCTTAGGCCC
COL3A1	GGTAGCCCTGGTGAGAGAGG	CGGAGCCCCTCTTTCTCCTT
COL4A3	GCCAATGAACATGGCTCCCA	GGTTTGGCTGTGAACGGCTA
FN1	AGCTTTGTGGTCTCCTGGGT	ACTGTGGCTCATCTCCCTCC
LAMB1	GAGGGTACTCGGGGGTCTTC	GTGTCCTGTTGGTCAGCTCG
TNC	CCACCCACTACACAGCCCAG	CCCCATCAGAGGTCATGTCA
MMP2	CCGTGTGAAGTATGGGAACG	TCATAGGATGTGCCCTGGAA
MMP9	CCAACTACGACACCGACGAC	GAAAGTGAAGGGGAAGACGC
CDH1	ACCATCCTCAGCCAAGATCC	TCAGGCACCTGACCCTTGTA
CDH2	ATCCCTCCAATCAACTTGCC	ATGAAACCGGGCTATCTGCT
ACTA2	GTGGGTGACGAAGCACAGAG	CATGGCTGGGACATTGAAAG
GAPDH	ACAACTTTGGTATCGTGGAA	AAATTCGTTGTCATACCAGG

COL1A1 : Collagen 1A1, COL1A2 : Collagen 1A2, COL3A1 : Collagen 3A1, COL4A3 : Collagen 4A3, FN1 : Fibronectin, LAMB1 : Laminin, TNC : Tenascin-C, MMP2 : Matrix metalloproteinase-2, MMP9 : Matrix metalloproteinase-9, CDH1 : E-cadherin, CDH2 : N-cadherin, ACTA2 : alpha smooth muscle actin, GAPDH : Glyceraldehyde 3-phosphate dehydrogenase.

Supplementary Table S2. Primer sequence for real-time PCR.

Gene	Primer sequence	
	Forward (5’-3’)	Reverse (3’-5’)
COL1A1	CGCTGGTTTCGACTTCAGCT	ACATTGGCATCATCAGCCCG
COL1A2	CTGGCCCCAATGGATTTGCT	CCGTTTTCACCCTTAGGCCC
COL3A1	GGTAGCCCTGGTGAGAGAGG	CGGAGCCCCTCTTTCTCCTT
COL4A3	GCCAATGAACATGGCTCCCA	GGTTTGGCTGTGAACGGCTA
FN1	AGCTTTGTGGTCTCCTGGGT	ACTGTGGCTCATCTCCCTCC
LAMB1	GAGGGTACTCGGGGGTCTTC	GTGTCCTGTTGGTCAGCTCG
TNC	TAGTGGTCAAGTGGGAGGGG	GCCTGTAAGCTTTTCCCAAGT
MMP2	CCCTTGTTTCCGCTGCATCC	GGTCCTGGCAATCCCTTTGTAT
MMP9	GAGCTGACTCGACGGTGATG	TCGCTGGTACAGGTCGAGTA
CDH1	GTCAGTTCAGACTCCAGCCC	GAGGCTGCGGCTCCAAG
CDH2	AGGCTTCTGGTGAAATCGCA	AGAGGCTGTCCTTCATGCAC
ACTA2	CCCGGGACTAAGACGGGAA	CTTACAGAGCCCAGAGCCAT
GAPDH	GCGTCTTCACCACCATGGAG	GACGAACATGGGGGCATCAG

COL1A1 : Collagen 1A1, COL1A2 : Collagen 1A2, COL3A1 : Collagen 3A1, COL4A3 : Collagen 4A3, FN1 : Fibronectin, LAMB1 : Laminin, TNC : Tenascin-C, MMP2 : Matrix metalloproteinase-2, MMP9 : Matrix metalloproteinase-9, CDH1 : E-cadherin, CDH2 : N-cadherin, ACTA2 : alpha smooth muscle actin, GAPDH : Glyceraldehyde 3-phosphate dehydrogenase.

Supplementary Figure 1. Primary HCC 118965 express Hep Par-1, AFP and albumin protein (A) and AFP and albumin mRNA (B).

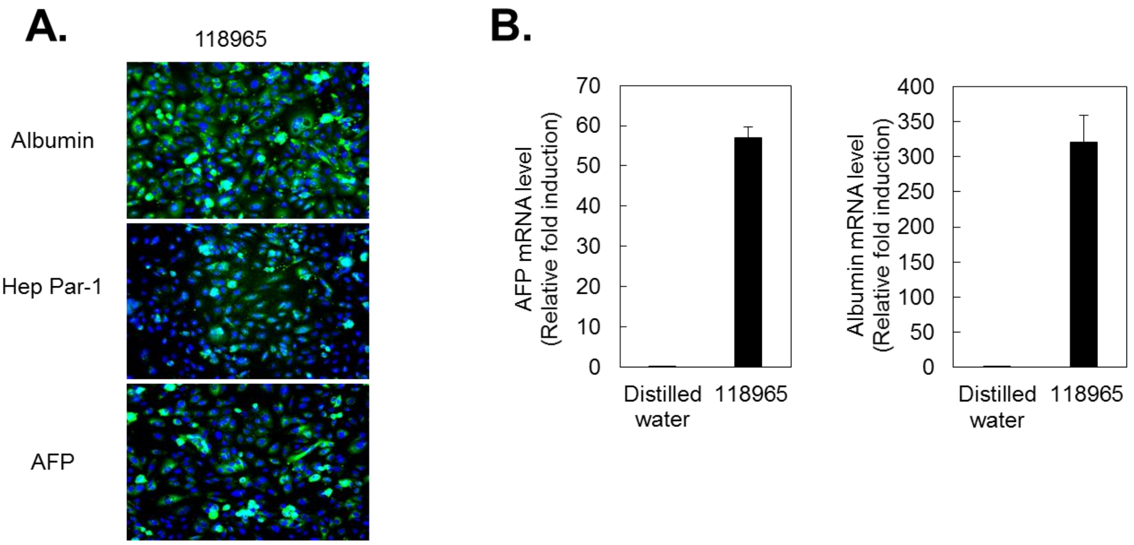

Supplement: Supplementary Information [file srep36750-s1.pdf]
